# Volunteer provision of long-term care for older people in Thailand and Costa Rica

**DOI:** 10.2471/BLT.16.187526

**Published:** 2017-05-26

**Authors:** Peter Lloyd-Sherlock, Anne Margriet Pot, Siriphan Sasat, Fernando Morales-Martinez

**Affiliations:** a School of Development Studies, University of East Anglia, Norwich, NR4 7TJ, England.; b Department of Ageing and Life Course, World Health Organization, Geneva, Switzerland.; c Faculty of Nursing, Chulalongkorn University, Bangkok, Thailand.; d Hospital Nacional de Geriatría y Gerontología, Universidad de Costa Rica, San José, Costa Rica.

## Abstract

**Problem:**

Demand for long-term care services for older people is increasing rapidly in low- and middle-income countries. Countries need to establish national long-term care systems that are sustainable and equitable.

**Approach:**

The Governments of Costa Rica and Thailand have implemented broadly comparable interventions to deploy volunteers in long-term home care. Both countries trained older volunteers from local communities to make home visits to impoverished and vulnerable older people and to facilitate access to health services and other social services.

**Local setting:**

Costa Rica and Thailand are upper-middle-income countries with strong traditions of community-based health services that they are now extending into long-term care for older people.

**Relevant changes:**

Between 2003 and 2013 Thailand’s programme trained over 51 000 volunteers, reaching almost 800 000 older people. Between 2010 and 2016 Costa Rica established 50 community care networks, serving around 10 000 people and involving over 5000 volunteers. Despite some evidence of benefits to the physical and mental health of older people and greater uptake of other services, a large burden of unmet care needs and signs of a growth of unregulated private services still exist.

**Lessons learnt:**

There is scope for low- and middle-income countries to develop large-scale networks of community-based long-term care volunteers. The capacity of volunteers to enhance the quality of life of clients is affected by the local availability of care services. Volunteer care networks should be complemented by other initiatives, including training about health in later life for volunteers, and investment in community long-term care services.

## Résumé

**Problème:**

La demande en services de soins de longue durée pour les personnes âgées augmente rapidement dans les régions à revenu faible et intermédiaire. Les pays doivent établir des systèmes nationaux qui soient pérennes et équitables pour les soins de longue durée.

**Approche:**

Les gouvernements du Costa Rica et de la Thaïlande ont lancé des initiatives assez comparables pour faire intervenir des personnes bénévoles dans les soins de longue durée à domicile. Dans ces deux pays, des bénévoles issus des communautés locales ont été formés pour rendre visite à des personnes âgées pauvres et vulnérables et les aider à accéder aux services de santé et d'aide sociale.

**Environnement local:**

Le Costa Rica et la Thaïlande sont des pays à revenu intermédiaire-supérieur avec une tradition fortement ancrée de services de santé à base communautaire, qu'ils cherchent désormais à étendre aux soins de longue durée des personnes âgées.

**Changements significatifs:**

Entre 2003 et 2013, le programme lancé en Thaïlande a permis de former plus de 51 000 bénévoles, qui sont intervenus auprès de quasiment 800 000 personnes âgées. Entre 2010 et 2016, le Costa Rica a constitué 50 réseaux de soins communautaires, qui se sont mis au service de près de 10 000 personnes, grâce à l'implication de plus de 5 000 bénévoles. Malgré de réelles répercussions positives sur la santé physique et mentale des personnes âgées et leur meilleure prise en charge par d'autres services, il existe un lourd fardeau de besoins en soins non satisfaits et des signes de développement de services privés qui ne sont pas réglementés.

**Leçons tirées:**

Il est possible pour les régions à revenu faible et intermédiaire de développer de larges réseaux de bénévoles pour aider à la prestation de soins de longue durée à base communautaire. Mais la capacité de ces bénévoles à améliorer la qualité de vie des bénéficiaires est affectée par la disponibilité locale des services de soins. La création de réseaux de bénévoles devrait être accompagnée d'autres initiatives, notamment pour former les bénévoles sur la santé du troisième âge et investir dans les services communautaires de soins de longue durée.

## Resumen

**Situación:**

La demanda de servicios de atención a largo plazo para personas mayores está aumentando rápidamente en países con ingresos bajos y medios. Los países necesitan establecer sistemas nacionales de atención a largo plazo que sean sostenibles y equitativos.

**Enfoque:**

Los gobiernos de Costa Rica y Tailandia han implementado intervenciones ampliamente comparables para desplegar voluntarios en la atención domiciliaria a largo plazo. Ambos países formaron a antiguos voluntarios de comunidades locales para realizar visitas a domicilio a personas mayores vulnerables y empobrecidas, y para facilitar el acceso a los servicios sanitarios y otros servicios sociales.

**Marco regional:**

Costa Rica y Tailandia son países con ingresos medios más altos y con fuertes tradiciones de servicios sanitarios de ámbito comunitario que actualmente están ampliando hacia el cuidado a largo plazo de personas mayores.

**Cambios importantes:**

Entre 2003 y 2013, el programa de Tailandia formó a 51 000 voluntarios y alcanzó casi 800 000 personas mayores. Entre 2010 y 2016, en Costa Rica se establecieron 50 redes de atención comunitaria, lo que permitió servir a cerca de 10 000 personas y contar con más de 5 000 voluntarios. A pesar de algunas pruebas sobre los beneficios en la salud mental y física de las personas mayores y un mayor uso de otros servicios, todavía existe una gran carga de necesidades de cuidados sin cubrir y señales de un crecimiento de servicios privados que no están regulados.

**Lecciones aprendidas:**

Los países con ingresos bajos y medios tienen un margen para desarrollar redes a gran escala de voluntarios para realizar cuidados a largo plazo y de ámbito comunitario. La capacidad de los voluntarios para mejorar la calidad de vida de los clientes se ve afectada por la disponibilidad local de los servicios de atención. Las redes de cuidado voluntario deberían complementarse con otras iniciativas, incluyendo la formación para voluntarios sobre la salud en las últimas etapas de la vida y la inversión en los servicios de atención a largo plazo de la comunidad.

## ملخص

**المشكلة:**

يتزايد الطلب على خدمات الرعاية طويلة الأمد لكبار السن بشكل سريع في البلدان منخفضة ومتوسطة الدخل. ولذلك تحتاج البلدان إلى إنشاء نظم وطنية للرعاية طويلة الأمد تراعي عوامل الاستدامة والإنصاف.

**الأسلوب:**

نفذت حكومتا كوستاريكا وتايلند تدخلات مماثلة على نطاق واسع لنشر المتطوعين في قطاع الرعاية المنزلية طويلة الأمد. وقام كلا البلدين بتدريب المتطوعين الأكبر سنًا من المجتمعات المحلية على القيام بزيارات منزلية للمسنين الفقراء والمستضعفين وتيسير سبل الحصول على الخدمات الصحية والخدمات الاجتماعية الأخرى.

**المواقع:**

**المحلية **تُعد كلاً من كوستاريكا وتايلند من بلدان الشريحة العليا من فئة الدخل المتوسط، كما أنهما ذواتا تقاليد قوية فيما يتعلق بالخدمات الصحية المجتمعية، والتي تمتد الآن إلى قطاع الرعاية طويلة الأمد لكبار السن.

**التغيّرات:**

**ذات** **الصلة **في الفترة ما بين عامي 2003 و2013، قام برنامج تايلند بتدريب أكثر من 51,000 متطوع ليتمكنوا بذلك من الوصول إلى 800,000 شخص من كبار السن **. ** وفي الفترة ما بين عامي 2010 و2016، أنشأت كوستاريكا 50 شبكة رعاية مجتمعية تخدم ما يقرب من 10,000 شخص وتضم أكثر من 5000 متطوع. وعلى الرغم من وجود بعض الأدلة على فوائد الصحة البدنية والعقلية لكبار السن وزيادة الاستفادة من الخدمات الأخرى، إلا أن هناك عبئًا كبيرًا من احتياجات الرعاية غير المستوفاة لا يزال قائمًا فضلاً عن وجود دلائل على تنامي الخدمات الخاصة غير الخاضعة للتنظيم.

**الدروس:**

**المستفادة ** يوجد مجال للبلدان منخفضة ومتوسطة الدخل لتطوير شبكات واسعة النطاق من متطوعي الرعاية المجتمعية طويلة الأمد. وتتأثر قدرة المتطوعين على تحسين نوعية حياة المستفيدين من الخدمة من خلال توفير خدمات الرعاية محليًا. وينبغي استكمال شبكات الرعاية التطوعية بمبادرات أخرى، بما في ذلك تدريب المتطوعين على الخدمات الصحية في المراحل اللاحقة من العمر، والاستثمار في خدمات الرعاية المجتمعية طويلة الأمد.

## 摘要

**问题:**

中低收入国家老年人对长期护理服务的需求不断攀升。 各国需建立持续且公正的国家长期护理体系。

**方法:**

哥斯达黎加政府及泰国政府已广泛实施了相当的干预以部署长期家庭护理方面的志愿者服务工作。 两国均对来自当地社区的老年志愿者进行了培训，以便他们能够对贫穷及弱势老年人进行家访，同时提升人们获取健康服务及其它社会服务的机会。

**当地状况:**

哥斯达黎加及泰国是中高收入国家，有着强大的以社区为基础的健康服务传统。现如今，这些健康服务已扩展到为老年人提供长期护理服务。

**相关变化:**

2003 至 2013 年期间，泰国计划培训了 51 000 位志愿者，覆盖近 800 000 名老年人。 2010 至 2016 年期间，在哥斯达黎加建立了 50 个社区护理网络，服务近 10 000 人，涉及 5000 多位志愿者。 除了一些表明对老年人身心健康有益的证据及其它服务的利用率提升以外，未满足的护理需求及不受监管的私人服务的增长迹象表明仍然存在巨大压力。

**经验教训:**

对中低收入国家来说，发展大规模以社区为基础的长期护理服务志愿者网络方面仍有相当大的空间。 志愿者提高客户生活质量的能力受当地护理服务的可用性的影响。 志愿者护理网络应通过其它举措进行补充，包括为志愿者提供与晚年健康相关的培训及对社区长期护理服务进行投资。

## Резюме

**Проблема:**

Спрос на услуги долгосрочного ухода за пожилыми людьми быстро растет в странах с низким и средним уровнем дохода. Странам необходимо создать национальные системы долгосрочного ухода, которые будут устойчивыми и справедливыми.

**Подход:**

Правительства Коста-Рики и Таиланда осуществили сопоставимые мероприятия по привлечению добровольцев к оказанию долгосрочной помощи на дому. Обе страны обучили добровольцев пожилого возраста из местных общин тому, как осуществлять визиты на дом к обездоленным и социально незащищенным пожилым людям и оказывать им помощь в доступе к медицинским и социальным услугам.

**Местные условия:**

Коста-Рика и Таиланд являются странами с доходом выше среднего уровня, с устойчивыми традициями служб здравоохранения на местном уровне, которые в настоящее время распространяются на долгосрочную помощь пожилым людям.

**Осуществленные перемены:**

В период между 2003 и 2013 годами в рамках программы в Таиланде были подготовлены более 51 000 добровольцев, благодаря чему помощь была оказана почти 800 000 пожилых людей. В период с 2010 по 2016 год в Коста-Рике было создано 50 сетей по оказанию помощи населению, которые обслуживают около 10 000 человек и задействуют более 5000 добровольцев. Несмотря на некоторые данные о пользе для пожилых людей в области физического и психического здоровья и более широком распространении других услуг, по-прежнему существуют большое бремя неудовлетворенной потребности в оказании медицинской помощи и признаки роста нерегулируемых частных услуг.

**Выводы:**

Для стран с низким и средним уровнем дохода имеется возможность развивать крупномасштабные сети добровольцев, работающих на долгосрочной основе на уровне общин. Способность добровольцев положительно влиять на качество жизни пожилых людей зависит от доступности на местном уровне служб по оказанию ухода. Сети добровольческой помощи должны быть дополнены другими инициативами, включая обучение добровольцев по вопросам здоровья в старческом возрасте и инвестирование в местные службы по оказанию долгосрочного ухода.

## Introduction

Long-term care refers to “the activities undertaken by others to ensure that people with a significant ongoing loss of capacity, or who are at risk of such a loss, can maintain a level of functional ability consistent with their basic rights, fundamental freedoms and human dignity.”^1^ The World Health Organization’s (WHO) *World report on ageing and health* in 2015 and related *Global strategy and action plan* recommend that every country should have a sustainable and equitable long-term care system for older people.^1^^,^^2^ To date, research on long-term care policies in low- and middle-income countries remains scarce.^2^^,^^3^ The *Global strategy and action plan* argues that volunteers may make a significant contribution to extending long-term care provision, especially in lower-resourced settings.^2^ We describe and compare two promising examples of long-term care models for older people: Costa Rica in Latin America and Thailand in south-east Asia. Despite the many differences between these two countries, both have implemented broadly comparable interventions to deploy volunteers in long-term care, building on established programmes of health volunteers.

## Local settings

Costa Rica and Thailand are selected here as examples of national long-term care schemes that make use of volunteers operating at the community level. These schemes developed independently of each other, with no direct support from WHO. Costa Rica and Thailand are upper-middle income countries, experiencing rapid economic, social and demographic transformation. In both, the proportion of the population aged 70 years or older is projected to double between the years 2000 and 2020 and is set to treble between 2000 and 2030 (Table 1).^4^

**Table 1:** Population aged 70 years or older in Costa Rica and Thailand: actual and projected, 2000–2030

Country and population	No. thousands (%)			
	Actual		Projected^a^	
	2000	2010	2020	2030
**Costa Rica**				
Total population	3 925 (100.0)	4 545 (100.0)	5 044 (100.0)	5 413 (100.0)
People aged ≥ 70 years	143 (3.6)	220 (4.8)	350 (6.9)	546 (10.1)
**Thailand**				
Total population	62 693 (100.0)	66 692 (100.0)	68 581 (100.0)	68 250 (100.0)
People aged ≥ 70 years	2 493 (4.0)	3 922 (5.9)	5 538 (8.1)	8 810 (12.9)

^a^Median variant fertility projections.

Source: United Nations,  2015.^4^

In 2002, Thailand published a national plan for older persons and the following year a care volunteer programme for older people was established.^5^ Since then, there has been rapid policy development, with an emphasis on community-based services to support volunteers as well as family caregivers.^6^ This builds on a previous initiative to train village health volunteers, initiated in 1997.

Costa Rica also has a well-developed infrastructure of primary health-care services and has pioneered several initiatives for older people, including a national long-term care network since 2010.^7^^,^^8^

## Approaches

In 2003 the Thai Government established the Home Care Service Volunteers for the Elderly programme for dependent older people.^9^^,^^10^ Home-care volunteers from local communities are trained for 18 hours by the Ministry of Social Development and Human Security. Volunteers classify older people’s level of dependency and identify individuals who do not have a family carer or are neglected. Each volunteer is responsible for around 15 older people in their respective communities. They provide a range of domiciliary care services, including assistance with eating, exercise and taking medicine, as well as liaising with local health workers, where appropriate. Volunteers receive around United States dollars (US$) 14 per month to cover local travel expenses.

In 2010 Costa Rica’s National Council for Older People established the Progressive Attention Network for Integral Elder Care. This nationwide scheme seeks to establish community-based long-term care networks with a focus on impoverished and highly vulnerable older people.^7^ Among other activities, the networks train retired teachers to act as unpaid community volunteer pensioners. This training is provided directly by the council and includes 3-day workshops, with a focus on geriatric health, integrated community care and identifying vulnerable older people. Applying a validated assessment tool, volunteers then identify vulnerable, dependent older people in their communities and report this information back to district health officers and the council. Volunteers are expected to make home visits at least once per month, or as the need arises. They provide a similar range of domiciliary services to those offered by the Thai volunteers, including assistance with nutrition, personal hygiene and taking medications. At the same time, the council is responsible for coordinating other local organizations, including nongovernmental organizations and government agencies, and for identifying local long-term care service priorities. Priorities vary, but typically include the provision of food supplements, free drugs and a US$ 100 monthly payment for domiciliary care. In 2015 the total cost of the programme was US$ 14 million. It is funded by a new, earmarked tax on the consumption of alcohol and tobacco, as well as a separate family allowance contribution, which includes a levy of 5% on the wages and salaries of public- and private-sector workers.

## Relevant changes

By 2013, Thailand’s home-care volunteers programme had been extended nationwide and had trained over 51 000 volunteers, reaching almost 800 000 older people, representing a potential starting point for a future extension of the service to all dependent older people.^6^ A 2014 survey of 3694 older people and 179 carers showed perceived benefits to the physical and mental health of older people, but the precise effects were not quantified.^11^ At the same time, evaluations from 498 local government representatives indicated that the perceived quality of services provided by volunteers has been variable.^6^^,^^11^ In the 2014 evaluation, only 33% of 76 provincial administrative districts reported that the care provided by volunteers was sufficient to meet older people’s long-term care needs.^11^ A common complaint from service receivers was that volunteers were not able to deal with common geriatric health problems, only general social issues such as social isolation. This has given rise to a new volunteer caregiver training initiative with a stronger focus on health services, set up by the Ministry of Public Health in 2016.

In Costa Rica, 50 community networks were established by 2016, operating in health districts in every province and serving around 10 000 people aged 65 years and older, and involving over 5000 volunteers. The programme is now in the process of being formally evaluated. Preliminary indications show that the network has led to a rise in the numbers of older people making use of different long-term care services, particularly those related to nutrition and companionship. However, in 2015 only 443 older people received payments to cover the costs of home care, which suggests a more limited reach. The programme appears to be well targeted, with around half of beneficiaries combining a high level of dependency, income poverty and other aspects of social vulnerability. Despite the rapid roll-out of the programme, evidence points to a large burden of unmet care needs and the growth of unregulated private services, providing paid domiciliary carers and nursing home facilities.^7^ Thailand has also seen a rapid expansion of unregulated, private long-term care services.^12^

## Lessons learnt

The main lessons learnt are summarized in Box 1. Comparing these two initiatives indicates that there is some potential for community volunteers to contribute towards long-term care provision for older people in lower-resource countries. This should, however, be only one element of a multipronged set of interventions integrating health and social care, as well as bridging formal and informal provision. Promoting long-term care volunteers is unlikely to be effective as a stand-alone policy.

Box 1 **Summary of main lessons learnt**

- Lower-resourced countries can develop large-scale networks of community-based volunteers providing long-term care for vulnerable older people, with relatively modest financial support.
- The capacity of volunteers to enhance the quality of life of clients is affected by the local availability of long-term care services.
- Volunteer care networks should be complemented by other initiatives, including training about health in later life for volunteers and investment in community long-term care services to build a system that is equitable and sustainable.

Establishing networks of volunteers to support care and facilitate access to local services permitted the governments of both countries to rapidly scale-up long-term care provision for vulnerable groups of older people. This was done with relatively modest financial support and was built on previous experiences of government-supported community health volunteering. Even with ambitious interventions, however, keeping pace with growing demand for long-term care is extremely challenging. As seen in Thailand, there is a need for volunteers to be trained in long-term care, integrating health and social care services for older people.

The capacity of volunteers to enhance the quality of life of clients was strongly affected by the local availability of other long-term care services. Distinguishing between mainstream health services and social services is not straightforward, and responsibility often cuts across different state agencies. This calls for a coordinated long-term care approach. In Costa Rica, this has been done at the local level through priority-setting by the government agency responsible. Thailand has taken a different approach by  delineating the responsibilities of different agencies in a national development plan in 2009.^13^

Finally, a rigorous, systematic evaluation of long-term care volunteer interventions is needed to identify good practice that may be transferable to other countries.
